# Engineering the sialome of mammalian cells with sialic acid mimetics

**DOI:** 10.1016/j.xpro.2023.102330

**Published:** 2023-06-28

**Authors:** Daniël L.A.H. Hornikx, Eline A. Visser, Venetia Psomiadou, Christian Büll, Thomas J. Boltje

**Affiliations:** 1 Department of Biomolecular Chemistry, Institute for Molecules and Materials, Radboud University, Nijmegen, the Netherlands; 2 Department of Synthetic Organic Chemistry, Institute for Molecules and Materials, Radboud University, Nijmegen, the Netherlands

**Keywords:** Cell culture, Flow Cytometry/Mass Cytometry, Chemistry

## Abstract

Mammalian glycans show a diversity in sialic acid capping, constituting the sialome. Sialic acids can be extensively modified chemically, yielding sialic acid mimetics (SAMs). Here, we present a protocol for detecting and quantifying incorporative SAMs using microscopy and flow cytometry, respectively. We detail steps for linking SAMS to proteins with western blotting. Lastly, we detail procedures for incorporative or inhibitory SAMs and how SAMs can be used for the on-cell synthesis of high-affinity Siglec ligands.

For complete details on the use and execution of this protocol, please refer to Büll et al.^1^ and Moons et al.^2^

## Highlights

• Introduction of SAMs into the mammalian sialoglycan biosynthesis pathway
• Specific inhibition of sialic acid capping
• Step-by-step instructions for the detection of Golgi-resident and cell surface sialoglycans
• Click chemistry protocol for sialoglycan functionalization of Siglec interactions

## Abstract (editor highlights)

Publisher’s note: Undertaking any experimental protocol requires adherence to local institutional guidelines for laboratory safety and ethics.

## Graphical abstract

**Figure:**
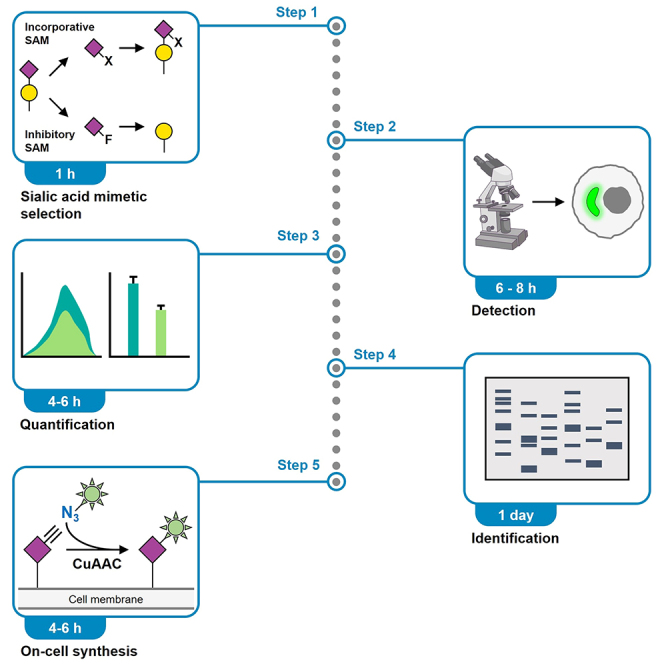

## Before you begin

### Sialic acid mimetic (SAM) selection

**Timing: 1 h**

The sialome consists of a diverse collection of sialylated glycans (sialoglycans). The structural complexity of the sialome arises from the type of sialic acid, glycosidic linkage, and glycoconjugate that carries the sialylated glycan. To probe sialic acid expression and their biological functions a large collection of SAMs has been synthesized with chemical modifications on one or more of the nine-carbon positions of the sialic acid backbone. Each SAM is designed to engineer and study various aspects of the sialome. SAMs generally enter the sialoglycan biosynthesis pathway, starting with formation of the respective CMP (cytidine monophosphate)-SAM nucleotide sugar by the enzyme CMAS (N-acylneuraminate cytidylyltransferase). CMP-SAMs are shuttled into the Golgi apparatus by the CMP-sialic acid transporter SLC35A1 and incorporation into glycans is catalyzed by the twenty sialyltransferase isoenzymes.

**Figure 1:**
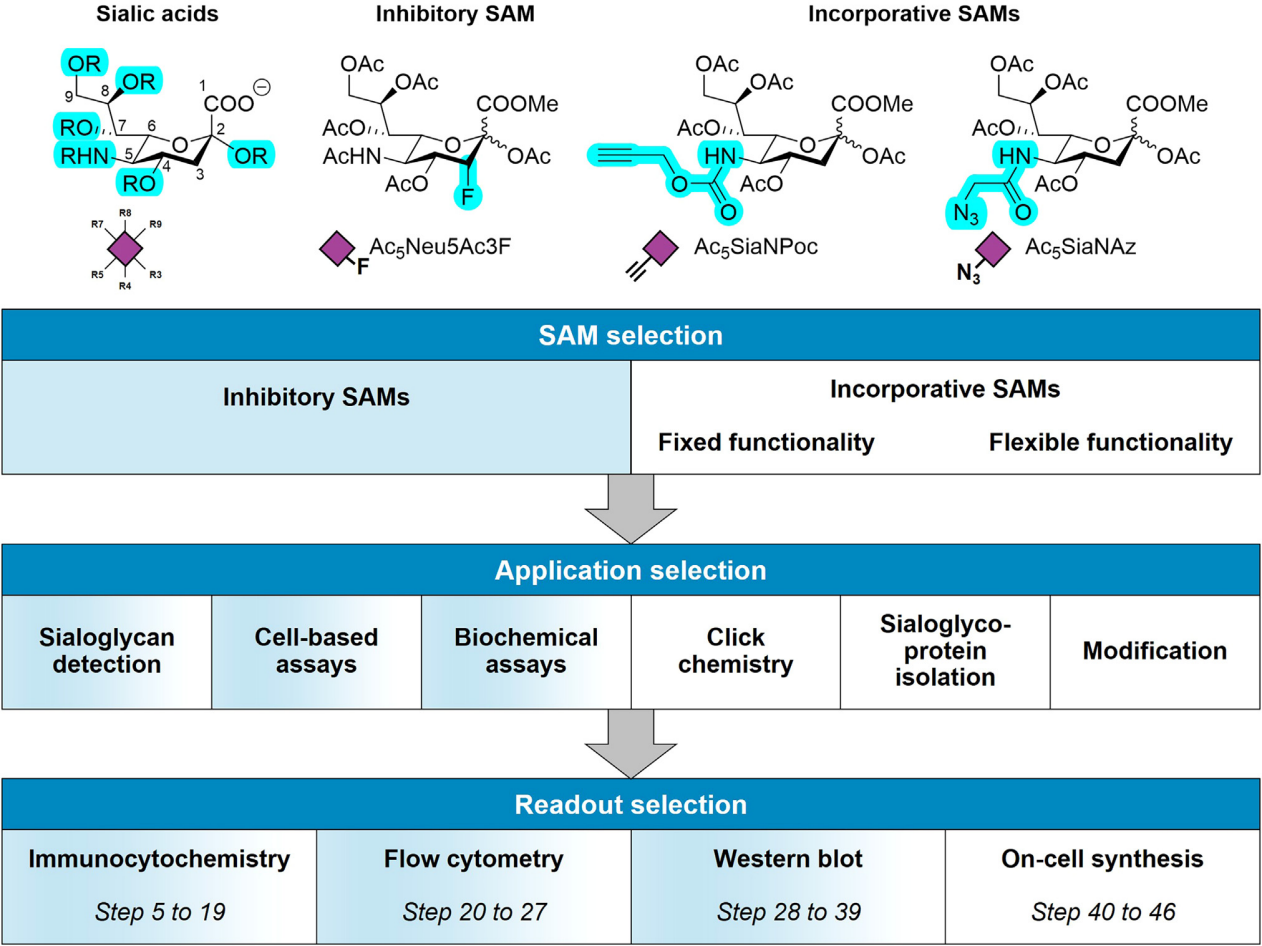
Chemical structure of sialic acid and the SAMs used in this protocol The scheme depicts step-wise selection of SAMs, applications, and readouts.

These steps describe how to select a SAM from the many different options (Figure 1).

Most SAMs can be grouped into two general classes: *Inhibitory* SAMs and *incorporative* SAMs.

*Inhibitory SAMs* interfere with one or more enzymes of the sialoglycan biosynthesis pathway and effectively halt the *de novo* production of sialoglycans.

*Incorporative SAMs* serve as substrates for the sialoglycan biosynthesis enzymes and are incorporated into sialoglycans. These can carry ‘fixed’ functional groups that cannot be further altered or ‘flexible’ groups that can be further modified once incorporated into sialoglycans on surface or secreted glycoproteins and glycolipids.

1. Selection of SAMs.
  a. Select inhibitory SAMs such as fluorinated SAMs that act as potent metabolic inhibitors of sialyltransferases, for example Ac_5_Neu5Ac3F, for deletion of sialic acid capping.^3^^,^^4^
  b. Select incorporative SAMs for the introduction of functional groups into sialic acids.

***Note:*** SAMs are available with either *fixed* functionality, such as 9-iodo-NeuAc,^5^ or with *flexible* functionality, such as the azide or alkyne-modified SAMs. Flexible SAMs such as Ac_5_SiaNAz and Ac_5_SiaNPoc, can be further modified using bioorthogonal chemistry.^6^

***Alternatives:*** Many different SAMs are available with one or more modifications designed for various applications.^2^^,^^5^^,^^7^^,^^8^^,^^9^^,^^10^^,^^11^

***Note:*** Sialic acids are negatively charged and their ability to cross the cell membrane is poor and no active uptake mechanism has been reported in mammalian cells. SAMs are therefore frequently peracetylated (e.g. Ac_5_SiaNAz) which allows them to pass the cell membrane *via* passive diffusion. Inside the cells, the acetyl groups are removed by esterases and the active SAM is released. Generally, the released acetyl groups have no significant effect on the cells when using SAM concentrations up to 100 μM.^12^ Note that non-acetylated SAM can be applied for metabolic engineering in some bacteria that express active sialic acid uptake mechanisms.^13^

***Note:*** Most SAMs have reversible effects that dilute with cell division and competing endogenous sialic acid biosynthesis. They can be supplemented to the culture medium for short and prolonged periods for temporal control of their effects.^14^

***Alternatives:****De novo* synthesis of sialic acids in mammalian cells starts from N-Acetylmannosamine (ManNAc) and is mediated by three enzymes (GNE, NANS, NANP). ManNAc derivatives such as the azide-containing Ac_4_ManNAz can also be used to introduce unnatural sialic acids into the sialoglycan biosynthesis pathway.^7^^,^^14^^,^^15^ However, ManNAc derivatives have to be tolerated by the three enzymes involved in the *de novo* synthesis, often have negative effects on cell proliferation, and they can be metabolized into other hexosamine sugars (e.g. GlcNAc).^7^ We therefore generally recommend SAMs for metabolic engineering of the sialome.

### Application selection

**Timing: 1 h**

SAMs allow to change the sialome of mammalian cells and are compatible with most cellular and biochemical assays. Therefore, multiple applications are available to apply SAMs in mammalian cells (Figure 1). These steps describe commonly used applications for selection.

2. If an inhibitory SAM is selected, e.g., Ac_5_Neu5Ac3F, the loss of sialic acid capping on cell surface glycans or secreted glycoproteins can be detected with glycan-binding proteins (GBPs).
  a. Select GBPs with specificity for sialoglycans to detect the loss of sialic acid-capping on cell surface or secreted glycans.
    ***Note:*** MALII (*Maackia amurensis* lectin II) and SNA-I (*Sambucus nigra* lectin I) are commonly used plant-derived GBPs to probe sialoglycan expression. They recognize α2-3 and α2-6-linked sialic acids to galactose, respectively.
  b. Cells treated with fluorinated SAMs are usually viable and can be applied in a wide range of cellular and biochemical assays to probe the role of sialoglycans herein.
  c. Select one of the many available adhesion, proliferation, migration, co-culture assays, or *in vivo* assays where many SAMs can be applied or any biochemical assay to probe the role of sialic acid-capping e.g., in protein function or glycan metabolism.
3. If an incorporative, flexible SAM is selected, e.g., Ac_5_SiaNPoc, a wide range of applications becomes available.
  ***Note:*** Applications include click chemistry-based strategies (copper(I)-catalyzed alkyne-azide-cycloaddition (CuAAC)) with large functionalization potential of incorporated azide/alkyne groups in sialoglycans by conjugation to alkyne/azide-containing molecules. Incorporative SAMs with fixed functionality can be directly assayed in cellular or biochemical assays.
  a. For visualization of sialoglycans in cellular compartments of live or fixed cells, select click chemistry-based conjugation of sialoglycans to azide/alkyne-modified fluorophores.
  b. For isolation/detection of sialoglycoproteins from the cell membrane or secretion, select click chemistry-based conjugation to azide/alkyne-biotin or other purification tags.
  c. For changing molecular interactions of sialoglycans with GBPs (e.g., Siglecs) or glycosidases, select click chemistry-based conjugation to azide/alkyne-modified small molecules that alter sialic acid – protein interactions.
    ***Note:*** Depending on their modifications, SAMs can be used for a wide range of applications compatible with mammalian cells not limited to the examples provided in this protocol.
    ***Note:*** Several GBPs are available with the ability to recognize sialoglycans or the loss thereof and can be applied to probe SAM-treated mammalian cells. For example, PNA (Peanut agglutinin) recognizes the beta-galactose units exposed after the loss of sialic acid capping.

### Readout selection

**Timing: 0.5 h**

Changes induced in the sialome of mammalian cells by SAMs can be assayed with several different methods and here we provide commonly used readouts for selection (Figure 1).

4. Select one of the following readouts to detect the effects of SAM.
  a. Select immunocytochemistry to probe changes in the sialome in live/fixed mammalian cells with structural information on the subcellular localization of sialoglycans. Samples can be analyzed by fluorescence-based microscopy.
  b. Select flow cytometry to obtain quantitative information on cell surface sialoglycan expression and binding levels to GBPs in cells treated with SAMs.
  c. Select western blot-based methods to yield information on how SAMs change the sialome on the level of individual surface and secreted sialoglycoproteins.

***Note:*** Several other readouts are available based on fluorescence, calorimetric assays or mass spectrometry.

### Preparation of SAM working stocks

**Timing: 1 h**

This step describes how SAMs provided as powder can be dissolved.

5. To prepare working stocks from SAMs in their powder form, dissolve SAMs to 100 mM in cell culture-grade dimethyl sulfoxide (DMSO) and mix/vortex until the powder is fully dissolved.

**CRITICAL:** DMSO is hazardous for the skin at levels of 0.1% or higher. Take proper safety precautions and wear proper personal protection equipment.

***Note:*** SAMs can be obtained from several commercial sources or are provided as community resource from several academic labs.

***Note:*** Aliquots of the working stocks can be stored at −20°C in Eppendorf tubes. Most dissolved SAMs including Ac_5_Neu5Ac3F, Ac_5_SiaNAz, and Ac_5_SiaNPoc are stable for at least one year and tolerate multiple freeze-thaw cycles.

***Alternatives:*** We recommend dissolving SAMs for working stocks in DMSO. Alternatively, SAMs can be dissolved in water, but their water solubility depends on their chemical composition and is different for every SAM. If needed, prepare high concentrations (e.g. 1 M) SAMs in DMSO and dilute stepwise in water until the desired concentration or saturation is reached.

#### Culturing of mammalian cells

**Timing: 1–2 weeks**

Sialoglycans are assembled by virtually all mammalian cells and therefore SAMs can be applied to most established mammalian cells lines as well as *ex vivo* cultures of human tissue-derived cells. These steps describe the preparation of human embryonic kidney cell (HEK293) and HeLa cell cultures, both adherent cell lines, and human T-lymphoblast Jurkat cell cultures, a suspension cell line.

6. Make preparations to start up cells for culture.
  a. Warm the culture medium and 1× PBS in a water bath at 37°C.
  b. Sterilize the cell culture hood and all required supplies with 70% ethanol.
7. Thawing of cryopreserved cells
  a. Fill 15-mL polystyrene tubes with 9 mL complete culture medium.
  b. Thaw cryovials containing HEK293 and Jurkat cells (∼1 M cells) from liquid nitrogen storage in a water bath at 37°C until 50% has thawed.
    ***Note:*** To reduce the chance of contamination, make sure to keep the O-ring and cap above the water surface. Thawing is a rapid process of approximately 2 minutes. The concentrations of DMSO in cryopreservation media can be toxic for the cells once thawed completely in a rapid manner, thus this should be prevented if possible.
  c. Sterilize the cryovial and transfer the cells from the cryovials into the tubes.
    ***Note:*** The leftover liquid can be pipetted out by hand and added to the 15-mL tube.
8. Centrifuge the tubes at 300 × *g* for 5 min at 20°C and remove supernatant by aspiration.
9. Seeding cells for culture
  a. Resuspend the cell pellets with 12 mL pre-warmed culture medium and transfer to T75 flasks.
  b. Place the flasks in a horizontal manner for adherent cells and vertical for suspension cells into a cell culture incubator at 37°C with 5% CO_2_ atmosphere.
  c. Culture the cells and check growth and adhesion daily. If required, change medium after 2–3 days.
  d. Passage the cells when they reach approx. 80% confluency (adherent cells) and ca. 2 × 10^6^ cells/mL (suspension cells).
10. Passaging of adherent cells
  a. At confluence ≥80%, remove the culture medium by aspiration from the flask.
  b. Wash the cells carefully with 10 mL 1× PBS for 30 s and aspirate the supernatant.
  c. Add 1 mL trypsin-EDTA and incubate for 2–5 min at 37°C until the cells detach.
  d. Add 9 mL fresh culture medium, resuspend the cells, and transfer to a 15 mL tube. Centrifuge at 300 × *g* for 5 min and remove supernatant.
  e. Resuspend the cells in 10 mL fresh pre-warmed culture medium and count viable cell numbers using trypan blue staining and the counting chamber.
  f. Seed required number of cells into the respective culture vessel. Passage at least 0.5 × 10^6^ cells back into a T75.
  g. Place cultures in an incubator at 37°C with 5% CO_2_ atmosphere.

**CRITICAL:** Some cell lines (e.g. HEK293) do not firmly adhere to culture plastic. Thus, liquid aspiration and addition steps must be performed by carefully adding liquids to the side of the flask and not directly onto the cells.

**CRITICAL:** The passage number of mammalian cells should not exceed 25 before metabolic engineering experiments, because they can go into senescence or alter their metabolism and characteristics with increasing passage number. Always check cell viability, growth, and morphology before using them in an experiment.

11. Passaging of suspension cells.
  a. Collect 10 μL cell suspension and count the cells to determine the density.
  b. If cells reach 2 × 10^6^ cells/mL, transfer cells into a polystyrene tube and centrifuge at 20°C, 300 × *g*, for 5 min.
  c. Remove the supernatant by aspiration and resuspend the cells in 10 mL fresh pre-warmed culture medium.
  d. Seed required number of cells into the respective culture dish containing at least 15 mL culture medium. Seed at least 1 × 10^6^ cells back into the main culture to maintain the cells.
  e. Place cultures in an incubator at 37°C with 5% CO_2_ atmosphere.

***Note:*** It can take up to two weeks before suspension cells enter exponential growth after recovery from cryopreservation. Growing them with medium containing 20% fetal-bovine serum (FBS) for the first week can facilitate recovery. Confluency of suspension cells is difficult to assess by microcopy and should be done by counting. Optimum growing density is 1.0–1.5 × 10^6^ cells/mL.

**CRITICAL:** Most SAMs are effective after 1–3 days of culture, because this time is typically required for the sialome turnover. When seeding cells, the density should allow them to grow for that period without overgrowing.

***Alternatives:*** In principle, all mammalian cell lines and fresh tissue-derived cells can be used for this protocol. Check the cell-type specific characteristics and growth conditions in literature or databases such as the American Type Culture Collection (ATCC) or the European Collection of Authenticated Cell Cultures (ECACC).

### Prepare the following

12. Pre-warm an incubator chamber or water bath to 37°C.
13. Pre-cool a plate/tube centrifuge to 4°C.
14. Initialize the CytoFLEX flow cytometer according to the manufacturer’s instructions. This cytometer contains a 488 laser and a 510/20 bandpass filter.
15. Setup the EVOS M5000 imaging system with ability to detect green, fluorescent dyes (excitation 490 nm, emission 510–525 nm) and DAPI (excitation 350 nm, emission 470 nm).

***Alternatives:*** Setup a flow cytometer or fluorescence microscope with similar parameters.

## Key resources table

**Table:** 

REAGENT or RESOURCE	SOURCE	IDENTIFIER
**Antibodies**		

Streptavidin, Alexa Fluor™ 488 conjugate (2 mg/mL)	Invitrogen™	Cat#S32354
Streptavidin, Alexa Fluor™ 647 conjugate (2 mg/mL)	Invitrogen™	Cat#S21374
eBioscience™ Streptavidin-PE conjugate (2 mg/mL)	Invitrogen™	Cat#12-4317-87
Goat anti-human IgG Fc, FITC conjugated (2 mg/mL)	Thermo Fisher Scientific	Cat#A18830
MALII, Biotin-conjugated (2 mg/mL)	Vector Laboratories Inc.	SKU#B-1265-1
Recombinant human Siglec-3/CD33 Fc chimera protein, CF (50 μg)	R&D Systems	1137-SL-050

**Chemicals, peptides, and recombinant proteins**		

Phosphate buffer saline (PBS), pH 7.2	Gibco™, Thermo Fisher Scientific	Cat#20012068
DMEM, high glucose, GlutaMAX™ Supplement, pyruvate	Gibco™, Thermo Fisher Scientific	Cat#31966047
RPMI 1640 Medium, GlutaMAX™ Supplement, HEPES	Gibco™, Thermo Fisher Scientific	Cat#52400025
Penicillin-streptomycin	Gibco™, Thermo Fisher Scientific	Cat#15140122
Fetal bovine serum (FBS)	Sigma-Aldrich	Cat#F0804
Trypsin-EDTA (0.5%)	Gibco™, Thermo Fisher Scientific	Cat#15400054
Ac_5_SiaNPoc	Synvenio	SV2474; CAS: NA
Ac_5_SiaNAz	Synvenio	SV2479 ; CAS: NA
Ac_5_Neu5Ac3F (P- Neu5Ac3F, P-3F_ax_-Neu5Ac)	Synvenio	SV2523; CAS: NA
Azide-PEG3-biotin conjugate	Sigma-Aldrich	Cat#762024; CAS: 875770-34-6
Biotin-PEG4-alkyne conjugate	Sigma-Aldrich	Cat#764213; CAS: 1262681-31-1
3-(2-azidoethyl)-1H-indole (Synthesized from 3-(2-Bromoethyl)indole)^1^ (L39-linker)	Sigma-Aldrich	Cat#376523; CAS: 3389-21-7
Dimethylsulfoxide (DMSO)	Sigma-Aldrich	Cat#D8418; CAS: 67-68-5
Poly-L-lysine hydrobromide	Sigma-Aldrich	Cat#P1524; CAS: 25988-63-0
Tween® 20 (polysorbate)	VWR	437082Q
Nonfat-dried milk, bovine	Sigma-Aldrich	M7409
Sodium chloride	Sigma-Aldrich	S9888; CAS: 7647-14-5
Tris base	Sigma-Aldrich	Cat#648310-M; CAS: 77-86-1
Glycine	Sigma-Aldrich	Cat#50046; CAS: 56-40-6
EDTA Titriplex III	Sigma-Aldrich	Cat#1.08418; CAS: 6381-92-6
Sodium dodecyl sulfate (SDS)	MPBio	SKU#04811033-CF; CAS: 151-21-3
4–15% Mini-PROTEAN® TGX™ Precast Protein Gels, 10-well, 30 μL	Bio-Rad	Cat#4561083
Ethanol	Sigma-Aldrich	Cat#1.00983; CAS: 64-17-5
Triton™ X-100	Sigma-Aldrich	Cat#T8787; CAS: 9002-93-1
cOmplete™ Protease Inhibitor Cocktail	Sigma-Aldrich	Cat#11697498001
Paraformaldehyde	Sigma-Aldrich	Cat#P6148; CAS: 30525-89-4
(+)-Sodium L-ascorbate	Sigma-Aldrich	Cat#A7631; CAS: 134-03-2
Copper(II) sulfate pentahydrate	Sigma-Aldrich	Cat#209198; CAS 7758-99-8
10× Carbo-free blocking solution	Vector Laboratories Inc.	SKU#SP-5040-125
Magnesium chloride hexahydrate	Sigma-Aldrich	Cat#M9272; CAS 7791-18-6
Calcium chloride dihydrate	Sigma-Aldrich	Cat#223506; CAS: 10035-04-8
Bovine serum albumin (BSA)	Sigma-Aldrich	Cat#A4503; CAS: 9048-46-8
DAPI	Sigma-Aldrich	Cat#D9542; CAS: 28718-90-3
Fluoromount™ Aqueous Mounting Medium	Sigma-Aldrich	Cat#F4680
PonceauS	Sigma-Aldrich	Cat#P7170; CAS: 6226-79-5
Trypan-blue 0.4%	Sigma-Aldrich	Cat#T8154; CAS: 72-57-1

**Critical commercial assays**		

Pierce BCA protein assay kit	Thermo Fisher Scientific	Cat#23225

**Experimental models: Cell lines**		

HEK293	ATCC	ATCC-CRL-1573
HeLa	ATCC	ATCC-CCL-2
Jurkat	ATCC	ATCC-TIB-152

**Software and algorithms**		

FlowJo VX	BD Biosciences	https://www.flowjo.com/
ImageJ Software	NIH (Schindelin et al.)^16^	https://imagej.net/software/ fiji/downloads version 2.9.0
GraphPad Prism (v8.0)	GraphPad Software Inc.	https://www.graphpad.com/scientific-software/prism/

**Other**		

Glass coverslips	VWR	BELC1943-00012

## Materials and equipment

**Table:** HEK293 and HeLa culture medium

Reagent (stock)	Final concentration	Amount
DMEM, high glucose, GlutaMAX™ Supplement	1×	445 mL
Fetal bovine serum (FBS)	10%	50 mL
Pen/Strep solution	1×	5 mL
**Total**	**N/A**	**500 mL**

Storage: 4°C for up to 4 months.

**CRITICAL:** Heat-inactivate the FBS at 56°C for 30 minutes before usage. Do not freeze heat-inactivated FBS, but store at 4°C for up to 4 months.

***Alternatives:*** Additional medium components can be added to the culture medium, depending on the cell line/type used.

**Table:** Jurkat culture medium

Reagent (stock)	Final concentration	Amount from stock
RPMI 1640 Medium, GlutaMAX™ Supplement, HEPES	1×	445 mL
Fetal bovine serum (FBS)	10%	50 mL
Pen/Strep solution	1×	5 mL
**Total**	**N/A**	**500 mL**

Storage: 4°C for up to 4 months.

**Table:** Poly-L-lysine coating solution

Reagent (stock)	Final concentration	Amount from stock
Poly-L-lysine hydrobromide	0.1 mg/mL	1 mg
1× PBS	1×	10 mL
**Total**	**0.1 mg/mL**	**10 mL**

Storage: 4°C for up to 1 month; −20°C for up to 1 year.

***Note:*** Poly-L-Lysine coating solution stored at 4°C or −20°C can be re-used ca. 10 times.

**Table:** Fixation buffer

Reagent (stock)	Final concentration	Amount from stock
Paraformaldehyde	4%	4 g
1× PBS	1×	80 mL, fill to 100 mL
NaOH	N/A	Used to adjust pH
**Total**	4%	**100 mL**

Storage: The solution can be aliquoted and frozen, or stored at 2°C–8°C for up to one month.

***Note:*** Add PFA powder to 1× PBS and stir at 60°C in fume hood. Add NaOH dropwise until PFA is dissolved and fill up to final volume with 1× PBS. Filter solution to remove particles.

**CRITICAL:** PFA is toxic! Take appropriate safety measures by wearing gloves and a lab coat and work under a fume-hood.

**Table:** Blocking buffer

Reagent (stock)	Final concentration	Amount from stock
BSA	5%	1 g
1× PBS	1×	20 mL
**Total**	**5%**	**20 mL**

Storage: 4°C, for up to 1 week.

**Table:** PBS-BSA buffer

Reagent (stock)	Final concentration	Amount from stock
BSA	1%	10 g
1× PBS	1×	1000 mL
**Total**	**1%**	**1000 mL**

Storage: 4°C. For long time storage, PBS-BSA can be supplemented with 0.02% Na-N_3_.

**Table:** Lectin staining solution

Reagent (stock)	Final concentration	Amount from stock
10× Carbo-free blocking solution	1×	5 mL
1 M MgCl_2_	1 mM	50 μL
1 M CaCl_2_	1 mM	50 μL
dH_2_O	N/A	44.9 mL
**Total**	**N/A**	**50 mL**

Storage: 3 months at 4°C.

***Note:*** Prepare stock solutions of 1 M MgCl_2_ and 1 M CaCl_2_ in MilliQ.

***Alternatives:*** 1% BSA in 1× PBS containing divalent ions is an alternative to carbo-free blocking solution.

**Table:** Permeabilization buffer

Reagent (stock)	Final concentration	Amount from stock
Saponin	0.1%	20 mg
1× PBS	1×	20 mL
**Total**	**N/A**	**20 mL**

Storage: Prepare fresh.

**Table:** Intracellular blocking buffer

Reagent (stock)	Final concentration	Amount from stock
BSA	5%	1 g
Saponin	0.1%	20 mg
1× PBS	1×	20 mL
**Total**	**N/A**	**20 mL**

Storage: Prepare fresh.

**Table:** Intracellular washing buffer

Reagent (stock)	Final concentration	Amount from stock
BSA	1%	1 g
Saponin	0.1%	100 mg
1× PBS	1×	10 mL
**Total**	**N/A**	**100 mL**

Storage: Prepare fresh.

**Table:** Sodium-L-ascorbate stock solution

Reagent (stock)	Final concentration	Amount from stock
Sodium-l-ascorbate	100 mM	20 mg
1× PBS	1×	1 mL
**Total**	100 mM	1 mL

Storage: −20°C (dry powder) in the dark. Prepare fresh before use.

**Table:** Copper sulfate stock solution

Reagent (stock)	Final concentration	Amount from stock
Copper sulfate pentahydrate	100 mM	25 mg
1× PBS	1×	1 mL
**Total**	100 mM	1 mL

Storage: Prepare fresh.

**Table:** Intracellular CuAAC solution

Reagent (stock)	Final concentration	Amount from stock
Saponin	0.1%	10 mg
100 mM Copper sulfate	0.5 mM	50 μL
100 mM Sodium-L-ascorbate	1 mM	100 μL
50 mM Azide-PEG_3_-Biotin	50 μM	10 μL
1× PBS	1×	9.84 mL
**Total**	**N/A**	**10 mL**

Storage: Prepare fresh (5–15 min before starting procedure).

***Alternatives:*** The CuAAC reaction buffer generates reactive oxygen (ROS) species that can damage cell membranes. To reduce the impact on cell viability, ROS-chelating agents e.g. l-histidine or THPTA can be added to the buffer.^17^

**CRITICAL:** Add sodium-l-ascorbate as last step before addition of the buffer to the samples. Sodium-L-ascorbate catalyzes Cu(II) into Cu(I) and for optimal reaction works best freshly prepared.

**Table:** Extracellular CuAAC solution

Reagent (stock)	Final concentration	Amount from stock
100 mM Copper sulfate	0.5 mM	50 μL
100 mM Sodium-L-ascorbate	1 mM	100 μL
50 mM Azide-PEG_3_-Biotin	50 μM	10 μL
1× PBS	1×	9.84 mL
**Total**	**N/A**	**10 mL**

Storage: Prepare fresh (5–15 min before starting procedure).

**Table:** Cell lysis buffer

Reagent (stock)	Final concentration	Amount from stock
1 M NaCl	150 mM	1.5 mL
1 M Tris-HCl pH 7.5	50 mM	0.5 mL
1 M EDTA	5 mM	50 μL
10% SDS	0.1%	100 μL
Triton-X100	1%	100 μL
50× Protease inhibitor cocktail	1×	200 μL
dH_2_O	N/A	7.55 mL
**Total**	**N/A**	**10 mL**

Storage: The hypotonic basis of the lysis buffer (NaCl, Tris-HCl, EDTA) can be stored at 20°C for up to 2 years. After addition of the other components, the lysis buffer can be stored at −20°C for up to 1 year. After preparation, keep on ice during the procedure.

**Table:** 10× Towbin buffer

Reagent (stock)	Final concentration	Amount from stock
Tris	25 mM	3 g
Glycine	192 mM	14.4 g
ddH_2_O	N/A	1000 mL
**Total**	**N/A**	**1000 mL**

Storage: Can be stored at 20°C for a for up to 2 years. pH should be around 8.6 ± 0.2

**Table:** SDS-PAGE running buffer

Reagent (stock)	Final concentration	Amount from stock
10× Towbin buffer	1×	100 mL
10% SDS	0.1%	10 mL
dH_2_O	N/A	890 mL
**Total**	**N/A**	**1000 mL**

Storage: Can be stored at 20°C for a for up to 2 years.

**Table:** Western blot transfer buffer

Reagent (stock)	Final concentration	Amount from stock
10× Towbin buffer	1×	100 mL
10% SDS	0.1%	10 mL
Ethanol	10%	100 mL
dH_2_O	N/A	790 mL
**Total**	**N/A**	**1000 mL**

Storage: Can be stored at 4°C for up to 2 weeks.

***Alternatives:*** The Western blot transfer buffer can be made with a final solution of 20% methanol, after which it can be reused 3–4 times.

**Table:** Membrane blocking buffer

Reagent (stock)	Final concentration	Amount from stock
BSA	2%	2 g
Nonfat-Dried milk, bovine	1%	1 g
1× PBS	1×	100 mL
**Total**	**N/A**	**100 mL**

Storage: Can be stored at 4°C for a week or for up to a year at −20°C.

**Table:** Membrane CuAAC solution

Reagent (stock)	Final concentration	Amount from stock
100 mM Copper sulfate	1 mM	0.3 mL
100 mM Sodium-L-ascorbate	2 mM	0.6 mL
50 mM Alkyne-PEG_4_-Biotin	50 μM	10 μL
1× PBS	1×	29.09 mL
**Total**	**N/A**	**30 mL**

Storage: Prepare fresh (5–15 min before starting procedure).

**Table:** PBS-T

Reagent (stock)	Final concentration	Amount from stock
Tween-20	0.05%	0.5 mL
1× PBS	1×	1000 mL
**Total**	**N/A**	**1000 mL**

Storage: 20°C for up to 1 year.

**Table:** On-cell Siglec ligand synthesis buffer

Reagent (stock)	Final concentration	Amount from stock
100 mM Copper sulfate	250 μM	25 μL
100 mM Sodium-L-ascorbate	500 μM	50 μL
50 mM L39-linker	100 μM	10 μL
1× PBS	1×	9 mL
**Total**	**N/A**	**10 mL**

Storage: Prepare fresh (5–10 min before addition to cells).

**Table:** Siglec-3 staining solution

Reagent (stock)	Final concentration	Amount from stock
Recombinant Siglec-3/CD33 Fc	2.5 μg/mL	4 μg
Goat anti-human IgG Fc, FITC conjugate	4.5 μg/mL	8 μg
BSA	1%	20 mg
1× PBS	1×	2 mL
**Total**	**N/A**	**2 mL**

Storage: Prepare fresh (20–30 min before addition to cells).

***Note:*** Mix the recombinant Siglec protein and anti-human Fc antibody and incubate 20 min at 4°C to form complexes. Pre-complexing forms Siglec multimers with high binding avidity.

## Step-by-step method details

### SAM treatment of mammalian cells

**Timing: 1–3 days**

These steps describe the treatment of mammalian cells with Ac_5_Neu5Ac3F that halts sialoglycan biosynthesis and the SAMs Ac_5_SiaNAz and Ac_5_SiaNPoc that allow introduction of clickable moieties into sialoglycans on surface and secreted glycoproteins and glycolipids.

1. Seed HEK293 (3 × 10^4^ cells/cm^2^), HeLa (2 × 10^4^ cells/cm^2^), or Jurkat cells (3 × 10^5^ cells/mL) 1 day before the start of the experiment into the desired culture dish format and cell density.
2. Dilute SAMs to a 10× concentration of the desired final concentration in culture medium.

***Note:*** For example, prepare 1 mL medium containing 1 mM SAM and add to 9 mL culture medium to yield 100 μM final concentration.

3. Add the SAM to the cells at a 1× final concentration and gently swirl the culture dish to equally distribute the SAM. For maximum effect (sialoglycan inhibition or incorporation) we recommend 100 μM SAM for HEK293, HeLa, and Jurkat cells.

**CRITICAL:** Always include a volumetric DMSO control sample treated with equal concentrations of DMSO to ensure that the observed effects are not mediated by this solvent.

4. Culture the cells for the desired amount of time and selected application.

***Note:*** 100 μM was found by us to be effective in most mammalian cell lines and types.^3^^,^^7^ However, the effective concentration of SAM differs per cell line/type and depending on the application different concentrations are applicable.

### Detection of cell-surface sialoglycans with GBPs

**Timing: 6–8 h**

These steps describe the detection of cell-surface sialoglycans with GBPs in adherent HeLa cells treated with Ac_5_Neu5Ac3F.

5. Preparation of glass coverslips for culture.
  a. Sterilize culture competent glass coverslips by dipping them into 70% ethanol for 30 s and wash thoroughly with 1× PBS.
  b. Place one coverslip/well into a 12-well plate and add 0.5 mL poly-L-lysine coating solution.
    ***Alternatives:*** Poly-L-Lysine is positively charged and enables attachment of net negatively charged cells to the cover slips preventing detachment during the staining procedure. Other reagents might also be suitable for coating.
  c. Incubate for 5 min at RT, remove the poly-L-lysine coating solution and thoroughly wash three times with 1 mL 1× PBS and aspirate all fluid.
    **CRITICAL:** Remove all free poly-L-Lysine to avoid its binding to cells, which might interfere with adherence.
6. Seed HeLa cells as described (preparation step 10) and add 100 μM of Ac_5_Neu5Ac3F, or DMSO control (step 2 and 3). Culture treated cells for up to 72 h.

**CRITICAL:** When adding the cells, ensure that the coverslip is at the bottom of the well and not floating to have cells growing only on one side.

7. Fix the cells.
  a. Aspirate the supernatant and wash the wells twice with 1 mL 1× PBS.
    ***Note:*** Supernatant can be stored for subsequent analysis of secreted glycoconjugates. Supernatant can be centrifuged at 300 × *g* for 5 min at 20°C to pellet debris and stored at −20°C.
  b. Remove the 1× PBS and add 0.5 mL fixation buffer and incubate for 10 min at RT.
    ***Note:*** Plates containing fixation buffer should not be placed back into the culture incubator, because the PFA might damage live cells in other well/plates due to cross contamination.
    **CRITICAL:** Do not pipet directly on the cells, but against the side of the well to avoid detaching the cells.
    ***Alternatives:*** When using a different method to fix cells on coverslips (methanol, acetone, or PEM buffer fixation), please check compatibility against a PFA control.
  c. Remove the fixation buffer and discard in hazardous compounds waste.
  d. Wash the wells carefully three times with 1 mL 1× PBS.
    **CRITICAL:** Wash thoroughly to remove all fixation buffer to prevent cross-linking of buffer proteins.
  e. Remove the 1× PBS and add 1 mL blocking buffer. Incubate the wells for 1 h at 20°C or >3 h at 4°C.
    **Pause point:** Coverslips with fixed cells can be stored in blocking buffer at 4°C for up to 2 months with 0.02% NaN_3_ added.
8. Stain the cells with biotinylated MALII.
  a. Wash the coverslip with 1 mL PBS-BSA and add 0.5 mL lectin staining solution containing 5 μg/mL biotinylated MALII.
  b. Incubate the samples for 1 h at 20°C or for >3 h at 4°C and wash three times with 1 mL PBS-BSA.

***Note:*** Some lectins require divalent ions (Ca^2+^, Mg^2+^) for binding and therefore should always be added to the lectin staining solution.

9. Detect bound MALII with immunofluorescence.
  a. Wash coverslips twice with PBS-BSA and incubate the samples for 30 min with 0.5 mL PBS-BSA containing 1 μg/mL streptavidin, Alexa Fluor™ 488 conjugate protected from light at 20°C.
    ***Note:*** Other secondary fluorescent streptavidin probes can be used, and samples can be stained with additional probes (e.g. antibodies) but ensure that these are compatible with the GBP.
  b. Wash the coverslips twice with 1 mL PBS-BSA and once with 1× PBS followed by incubation with 0.5 mL 1 μg/mL DAPI in 1× PBS for 10 min at 20°C in the dark.
10. Wash the coverslips once with 1 mL 1× PBS and mount them with mounting medium onto a microscopy slide and seal the edges of the coverslip with transparent nail polish.
11. Acquire representative images using the EVOS M5000 imaging system or alternative fluorescence microscopes.

### (Sub)cellular localization of alkyne-tagged sialoglycans

**Timing: 6–8 h**

These steps describe how alkyne-tagged sialoglycans can be detected in the Golgi apparatus using bioorthogonal chemistry.

12. Seed HeLa cells on poly-L-lysine coverslips in 12-wells (step 5) and culture them for 2 h in the presence of 100 μM Ac_5_SiaNPoc or DMSO control as described in preparation step 10.
13. Fix the cells as described in step 7 and permeabilize the cells by adding 0.5 mL permeabilization buffer for 10 min at RT.

**Pause point:** Coverslips with fixed cells can be stored in blocking buffer at 4°C for up to 2 months with 0.02% NaN_3_ added. This can only be done before permeabilization of the cells.

***Note:*** Additional probes to stain extracellular targets can be used after fixation and before adding the permeabilization buffer.

14. Remove the permeabilization buffer and add 1 mL intracellular blocking buffer for 60 min at 20°C.
15. Wash the coverslips three times with 1 mL intracellular washing buffer and incubate with intracellular CuAAC solution for 45 min at 37°C.

**CRITICAL:** Thoroughly wash the samples with 1× PBS to remove all free protein from the blocking step, because free proteins can bind the copper and reduce the reaction efficiency.

16. Aspirate the CuAAC solution and wash the samples three times with 1 mL intracellular washing buffer.
17. Incubate the samples with intracellular washing buffer containing 1 μg/mL streptavidin, Alexa Fluor™ 488 conjugate protected from light at 20°C .

***Note:*** Probes, e.g. antibodies against intracellular targets, can now be used for additional staining.

18. Wash three times with 1 mL intracellular washing buffer and once with 1× PBS. Perform DAPI staining and mounting as described in steps 9 and 10.

**Pause point:** Sealed samples can be stored in the dark at 4°C for up to 3 years.

19. Acquire representative images using the EVOS M5000 system or alternative fluorescence microscopes.

### Flow cytometry analysis of sialome engineering

**Timing: 4–6 h**

These steps describe how the dose-dependent effects of Ac_5_Neu5Ac3F and Ac_5_SiaNPoc on the sialome of Jurkat cells can be quantified by flow cytometry using MALII and click chemistry-based detection of sialoglycans, respectively.

20. Start with Jurkat cells growing for 3 days in a 96-wells U-bottom plate (ca. 0.2–0.4 × 10^6^ cells/well) in the presence of 0–256 μM of Ac_5_Neu5Ac3F and Ac_5_SiaNPoc and equal volumes of DMSO vehicle control.
21. Wash the cells three times
  a. Centrifuge the plate in a plate centrifuge at 500 × *g* for 3 min at 4°C.
  b. Remove the supernatant by ‘flicking’ the plate.
    **CRITICAL:** Before ‘flicking’ of the plate, make sure that a pellet is visible after centrifugation to avoid loss of cells.
  c. Resuspend the cells in 150 μL PBS-BSA.
22. Stain membrane sialoglycans with biotinylated MALII lectin.
  a. Resuspend the cell pellet in 100 μL PBS-BSA containing 5 μg/mL biotinylated MALII and incubate the samples for 45 min at 4°C.
  b. Centrifuge the plate at 500 × *g* for 3 min at 4°C and remove the supernatant by ‘flicking’ and wash twice with 150 μL PBS-BSA by centrifugation.
23. Label alkyne-tagged surface sialoglycans by click chemistry.
  a. Resuspend the cell pellets in 100 μL extracellular CuAAC solution and incubate for 20 min at 37°C.
  b. Centrifuge the plate at 500 × *g* for 3 min at 4°C and remove the supernatant by ‘flicking’ and wash three times with 150 μL PBS-BSA buffer by centrifugation.
24. Resuspend the cell pellets in 100 μL PBS-BSA containing 1 μg/mL streptavidin-PE conjugate and incubate for 20 min at 4°C protected from light.
25. Wash the samples three times with 150 μL PBS-BSA by centrifugation.

***Alternatives:*** Cells can be stained with other probes/dyes (viability, surface marker), provided they do not interfere with MALII binding and are compatible with the selected fluorophores.

26. Resuspend the cell pellets in 100 μL PBS-BSA and keep at 4°C protected from light.

***Note:*** Immediately before the measurement, resuspend the cells by pipetting or using build-in shaking options form the flow cytometer to ensure that the cells are in solution.

27. Acquire the samples using the CytoFlex flow cytometer plate-reader function or a flow cytometer with similar parameters. For data analysis go to the quantification and data analysis step.

***Alternatives:*** Other flow cytometer systems with similar parameters can be used for analysis.

### Detection of sialoglycoproteins by western blot

**Timing: 1 day**

These steps describe how alkyne-tagged sialoglycoproteins can be detected in total cell lysates and supernatant derived from HEK293 cells using western blotting.

28. Start with HEK293 cells growing in a 6-well plate (ca. 1 × 10^6^ cells/wells) for 3 days in the presence of 100 μM Ac_5_SiaNAz or equal concentrations of DMSO (step 3).
29. Take off the supernatant
  a. Collect the supernatants into 1.5 mL microcentrifuge tubes.
  b. To remove cells, centrifuge the supernatant tubes at 300 × *g* for 5 min and transfer supernatant into clean 1.5 mL microcentrifuge tubes.

***Alternatives:*** Different concentrations of SAM and different time points can be chosen. Keep in mind that a certain incorporation time, min. 8–16 hours, is required to accumulate detectable amounts of alkyne label in surface and secreted sialoglycoproteins.

30. Lyse the cells.
  a. Gently wash the adherent cells twice with 1 mL 1× PBS (RT) and aspirate the supernatant.
  b. Add 400 μL ice-cold cell lysis buffer per well and detach the cells by repeated vigorous pipetting (hold plate at a 45° angle) or scraping with the pipette tip or cell scraper.
  c. Check that all cells are detached with a light microscope at 4× or 10× magnification.
  d. Hold the plate at a 45° angle and transfer the samples into 1.5 mL microcentrifuge tubes and incubate them on ice for 1 h and vortex for 10 s after 30 min.
31. Vortex the sample tubes and centrifuge in a microcentrifuge at high speed (>10.000 × *g*) for at least 10 min at 4°C. Transfer the supernatant containing soluble sialoglycoproteins to a clean 1.5 mL microcentrifuge tube.

***Note:*** A small white pellet consisting of the insoluble components (e.g. membranes, DNA) should be visible in the tube. This can be used for subsequent analysis of insoluble molecules, by resuspending it in 2% SDS.

**Pause point:** Collected supernatants and cell lysates can be stored at −20°C for several years.

32. Determine the protein concentration in the cell lysates using a BCA Protein Assay Kit following the manufacturers instruction.
33. Load 30–50 μg of cell lysate per sample on a protein gel and run the gel 30 min at 60 V to run the samples out the slots and 60 min at 130 V in SD-PAGE running buffer to separate proteins by size. Transfer the proteins onto a nitrocellulose membrane for at least 60 min at 275 mA/100 V using ice cold 1× transfer buffer.

***Optional:*** Directly after transfer, stain the nitrocellulose membrane with Ponceau S solution to detect the blotted lysates and confirm equal loading of protein. Rinse the blot in dH_2_O once and submerge the blot in Ponceau S solution in a fume hood. Incubate the blot for at least 5 min at 20°C gently shaking (30–50 rpm), after which the Ponceau S solution can be removed. Rinse the blot in dH_2_O till background of blot appears clear and acquire the image of the membrane.

**Pause point:** membranes can be dried and stored for at least 3 years at 20°C.

34. Place the membrane into a clean incubation tray with the protein side facing upwards and add 30 mL membrane blocking buffer and incubate shaking for 1 h at 20°C or >4 h at 4°C.
35. Discard the blocking buffer and wash the membrane three times for 5 min with 30 mL 1× PBS and once with 30 mL demi water shaking at 20°C.

**CRITICAL:** Thoroughly wash the membrane to remove all free protein from the blocking step, because free proteins can bind the copper and reduce the reaction efficiency.

36. Incubate the membrane in 30 mL membrane CuAAC solution for 45 min at 37°C shaking.
37. Discard the click reaction buffer and wash the membrane four times for 5 min with 20 mL PBS-BSA shaking at RT.
38. Transfer the membrane into a 50 mL tube and add 4 mL PBS-BSA containing 1 μg/mL Streptavidin, Alexa Fluor™ 647 conjugate and incubate on a tube roller for 1 h at 20°C or >4 h at 4°C protected from light.

***Alternatives:*** Membranes can be stained with other probes and antibodies and secondary antibodies conjugated to compatible fluorophores to co-localize for example the sialoglycan and a specific protein signal.

39. Wash the membrane four times for 5 min with 20 mL PBS-T at 20°C and image the membrane with a fluorescent membrane imaging system.

### On-cell synthesis of high affinity Siglec ligands

**Timing: 4–6 h**

Incorporative SAMs with azide or alkyne tags allow further chemical modification with click chemistry once incorporated into sialoglycans. These steps describe a method to chemically modify sialic acids at the cell surface of living cells, rendering them into high-affinity Siglec ligand expressing cells (HASLECs).^1^

40. Culture suspension Jurkat cells for 72 h with 100 μM Ac_5_SiaNPoc or DMSO control.
  a. Distribute the cells into a 96-well V-bottom plate with 0.1–0.2 × 10^6^ cells/well.
  b. Centrifuge the plate in a plate centrifuge for 3 min, 500 × *g*, at 4°C and discard the supernatant by flicking the plate. Wash twice by resuspension in 150 μL 1× PBS by centrifugation and flicking.

**CRITICAL:** Thoroughly wash the cells to remove all medium proteins, because free proteins can bind the copper and reduce the reaction efficiency.

41. Remove the 1× PBS from the last wash step and resuspend the cells in 100 μL on-cell Siglec ligand synthesis buffer. Incubate the plate for 20 min at 37°C.

***Note:*** Add the reaction buffer also to DMSO-treated cells to determine the background.

42. Centrifuge the plate with 500 × *g* for 3 min at 4°C and discard the supernatant by flicking of the plate. Wash twice with 1× PBS-BSA by centrifugation and removing the supernatant by flicking.
43. Resuspend the cells in 100 μL Siglec-3 staining buffer and incubate for 1 h at 4°C protected from light.

***Note:*** Incubate one or more wells with PBS-BSA without recombinant Siglec-3/CD33 Fc chimera as negative control.

44. Centrifuge the plate with 500 × *g* for 3 min at 4°C and discard the supernatant by flicking of the plate. Wash twice with 150 μL PBS-BSA by centrifugation and removing the supernatant by flicking.
45. Resuspend the cell pellets in 100 μL PBS-BSA and pipet up and down to create single cell suspensions.

***Alternatives:*** Cells can be stained with other probes/dyes (viability, surface marker), provided they do not interfere with Siglec binding and are compatible with the selected fluorophores.

***Note:*** Immediately before and during the measurement, resuspend the cells by pipetting or using build-in shaking options to ensure that the cells are in solution.

46. Acquire the samples using the CytoFlex flow cytometer plate-reader function or a flow cytometer with similar parameters. For data analysis go to the quantification and data analysis step.

***Alternatives:*** As an alternative to CuAAC, strain-promoted azide-alkyne cycloaddition (SPAAC) can be used for the bioorthogonal chemistry. This reaction is spontaneous and does not require copper and is more suitable for live-cell modifications. However, the copper-catalyzed azide and alkyne L39 conjugation forms a triazole that maybe required for high-affinity Siglec-3 binding.

## Expected outcomes

**Figure 2:**
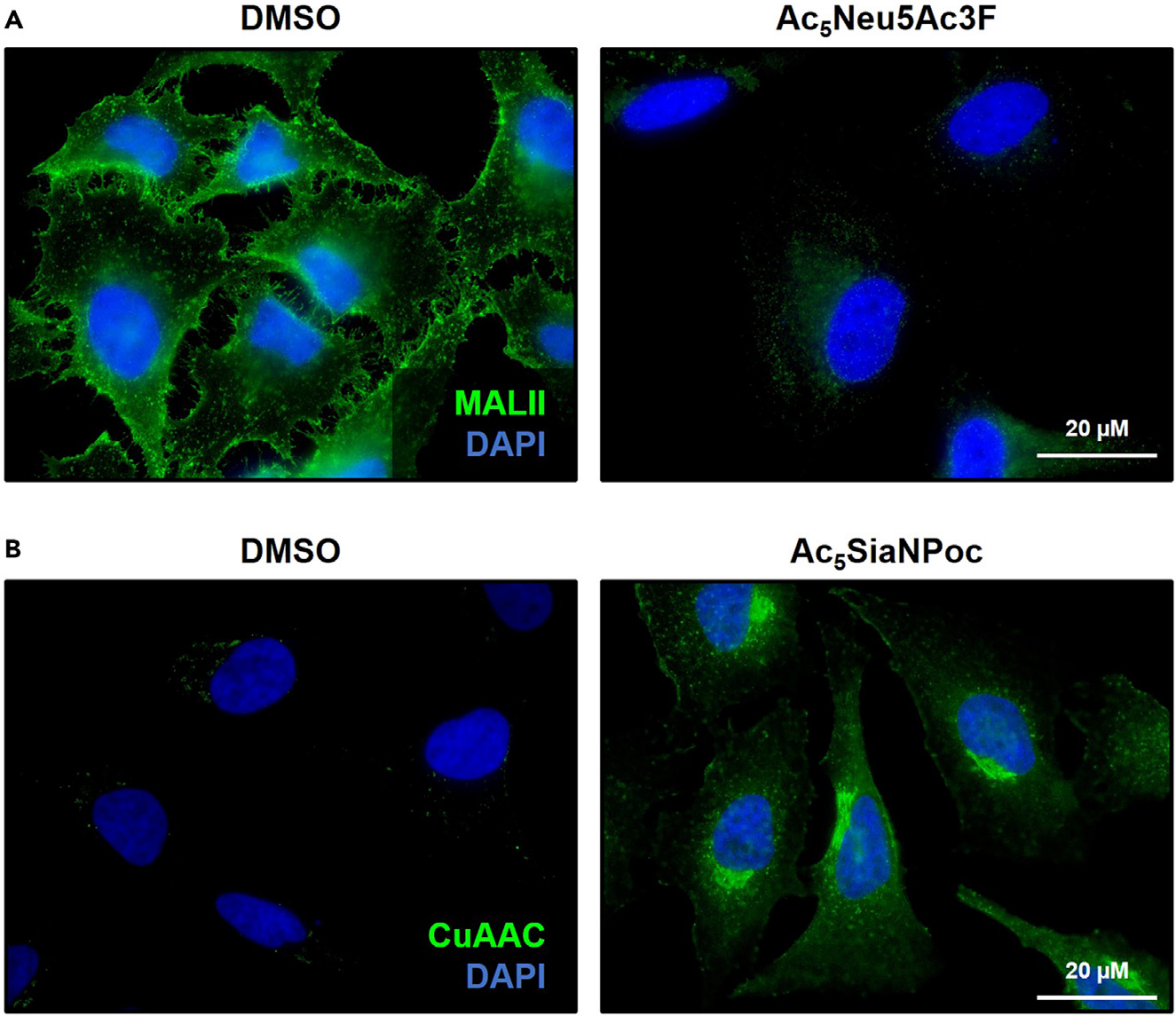
Detection of sialoglycans with immunocytochemistry (A) Representative fluorescence microscopy image of HeLa cells treated with DMSO and Ac_5_Neu5Ac3F for 3 days stained with MALII and DAPI. (B) Images show HeLa cells incubated for 2 h with DMSO or Ac_5_SiaNPoc followed click chemistry-based detection of intracellular alkyne-tagged sialoglycoproteins visible in the Golgi apparatus.

**Figure 3:**
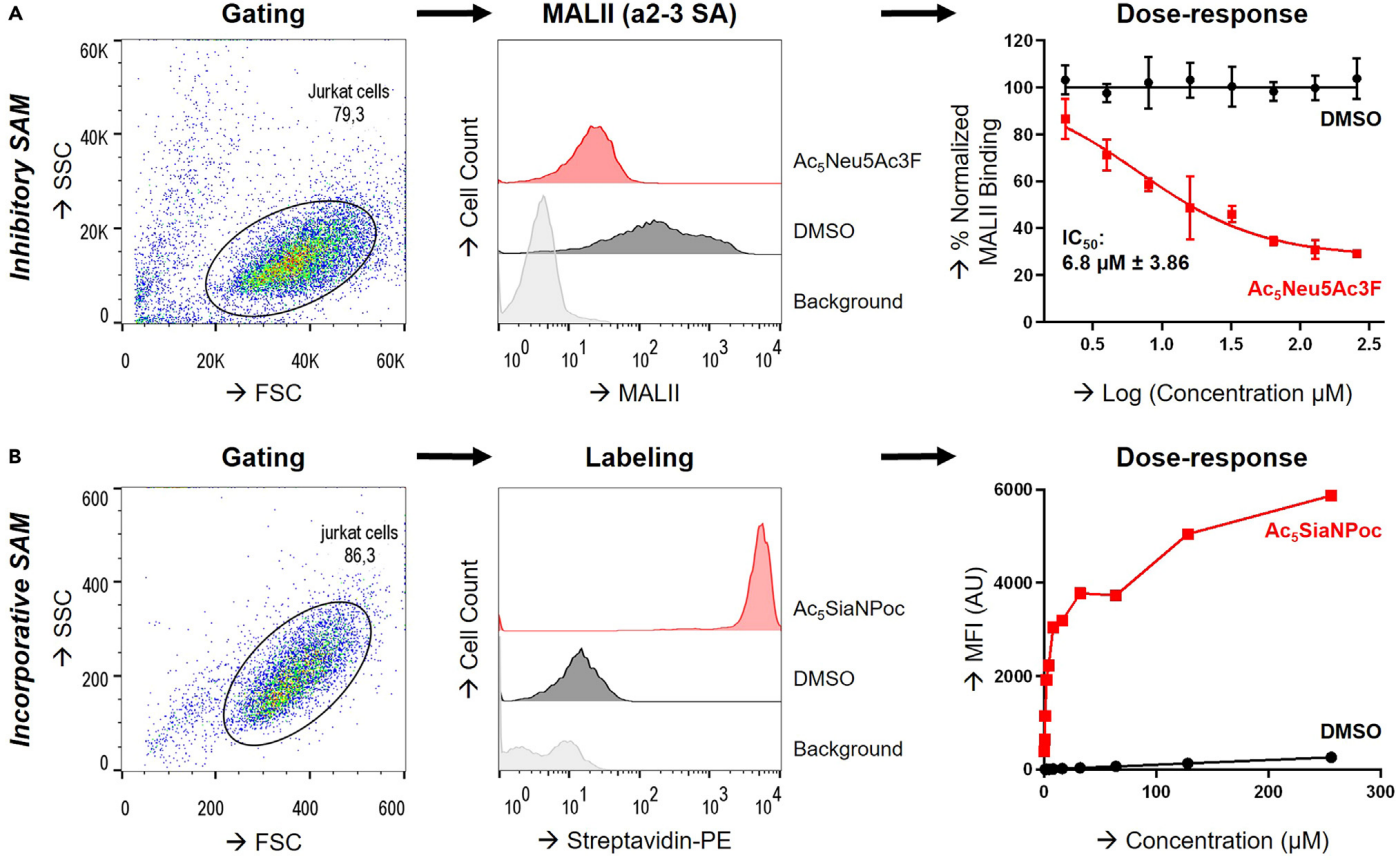
Flow cytometry-based quantification of SAM effects on the sialome (A and B) Jurkat cells were cultured for 3 d with increasing concentrations of DMSO, Ac_5_Neu5Ac3F, or Ac_5_SiaNPoc and labeled with MALII (A) or tagged using CuAAC (B). Gating on Jurkat cells based on the forward scatter (FSC) and side scatter (SSC) is shown (left) and histograms show fluorescence signal at 128 μM concentration (middle). Dose-response curves for both SAMs and the EC50 value for Ac_5_Neu5Ac3F are depicted (right). Error bars present SD (n=3).

**Figure 4:**
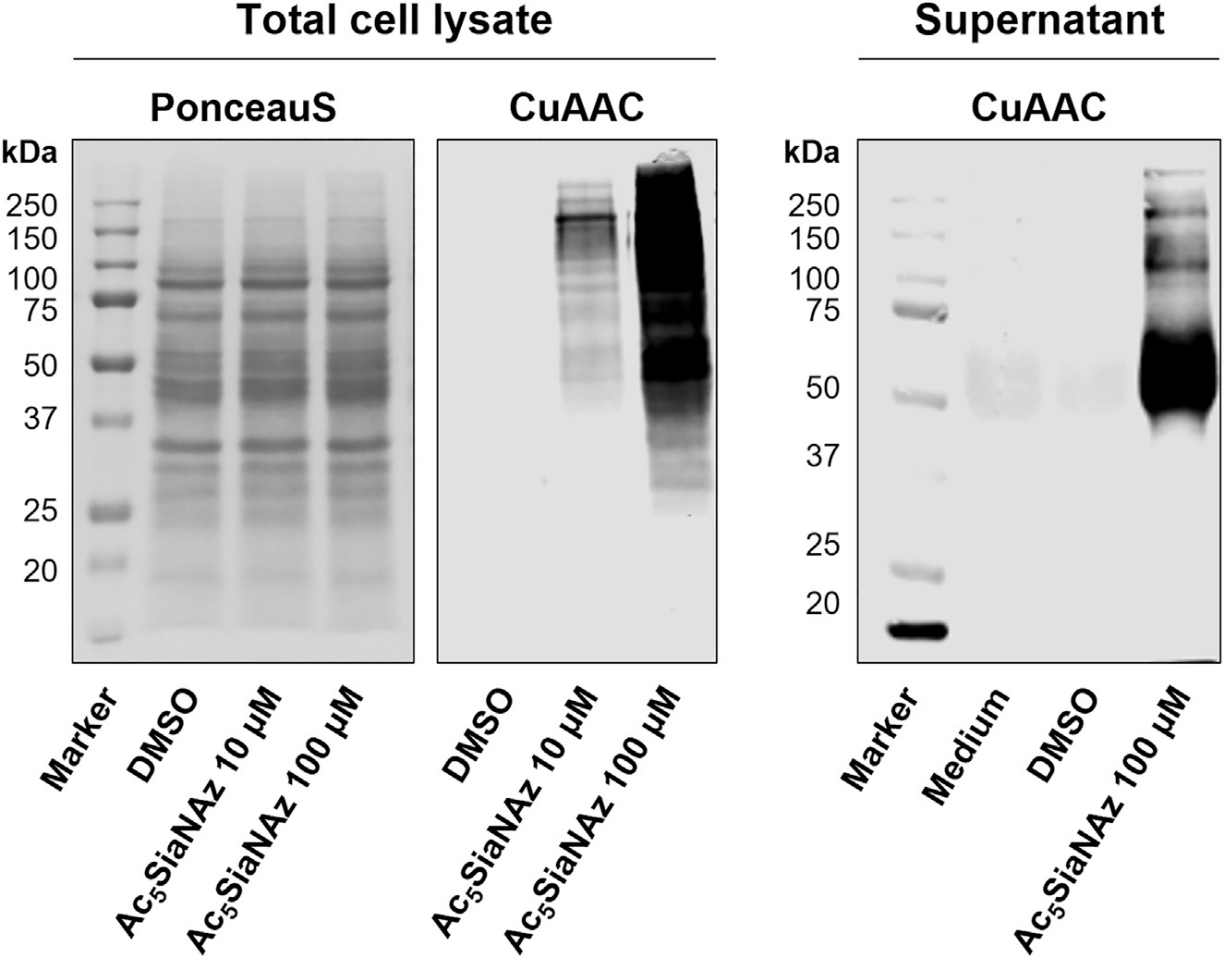
Western blot detection of sialoglycoproteins HEK293 cells were incubated for 3 d with DMSO, 10 μM or 100 μM Ac_5_SiaNAz and cell lysates and culture supernatant were subjected to western blot analysis. PonceauS staining shows equal protein loading and CuAAC visualizes sialoglycoproteins.

**Figure 5:**
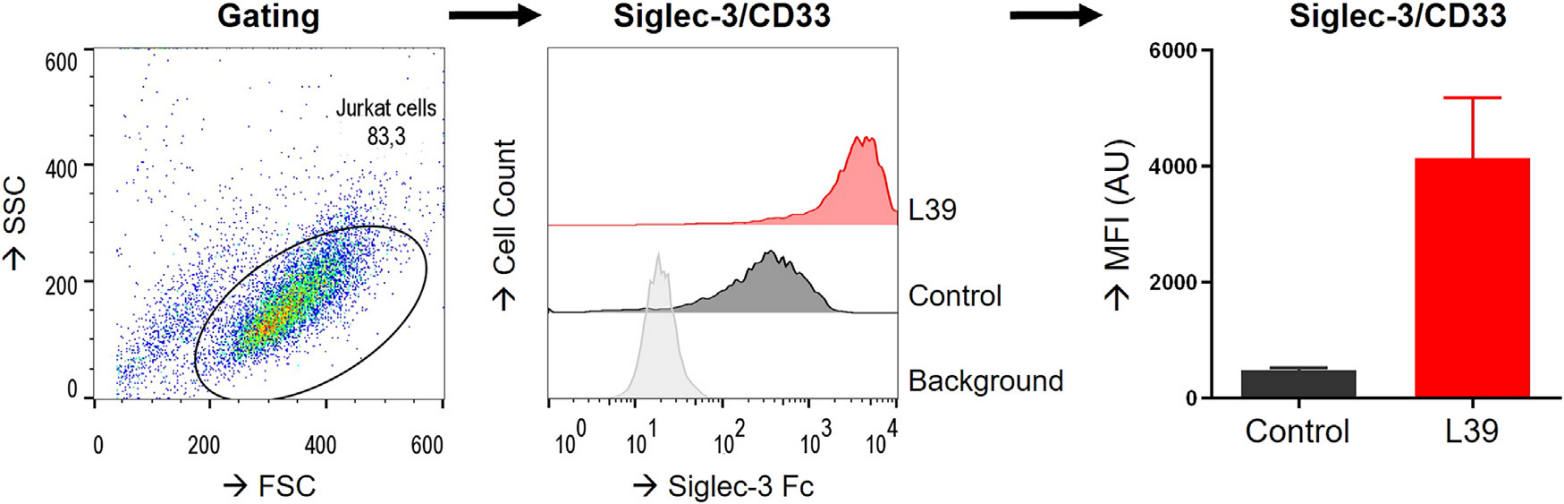
On-cell synthesis of Siglec-3/CD33 ligands Jurkat cells treated with 100 μM Ac_5_SiaNPoc for 3 d were functionalized with alkyne ligand L39 and stained with recombinant Siglec-3/CD33. Gating strategy and bar diagram with error bars indicating SD (n=3) is shown.

Our protocol describes the application of SAMs to engineer the sialome of mammalian cells. The effects of the inhibiting SAM Ac_5_Neu5Ac3F that interferes with sialoglycan biosynthesis can be detected by reduced binding of MALII lectin to the cell surface of HeLa cells with fluorescence microscopy (Figure 2A). The incorporative SAM Ac_5_SiaNPoc is installed in the sialoglycans of HeLa cells within 2 h allowing detection after click reaction in the Golgi apparatus fluorescence microscopy (Figure 2B). Flow cytometry-based detection of cell surface bound MALII or click-chemistry-labeled sialoglycans forms a quantitative readout to determine dose-response curves that inform on the degree of sialoglycan synthesis inhibition or incorporation, respectively. Representative gating and quantification is shown for Jurkat cells treated with different concentration of Ac_5_Neu5Ac3F or Ac_5_SiaNPoc (Figure 3). Sialoglycoproteins in total cell lysate or in culture supernatant of HEK293 cells can be detected by incorporative SAMs with clickable groups and western blot analysis (Figure 4). Lastly, a representative example for on-cell synthesis of a high-affinity Siglec-3/CD33 ligand (L39) in HEK293 cells treated with Ac_5_SiaNPoc and analysis by flow cytometry is shown (Figure 5).

## Quantification and statistical analysis

**Timing: 1–2 h**

This part describes quantification of flow cytometry data derived from GBP binding or click chemistry-based fluorescent labeling of surface sialoglycans.

1. Mean fluorescence intensity (MFI) from flow cytometry data were derived using FlowJo. Live cells were gated based on the forward (FSC) and side scatter (SSC) and their fluorescence intensity values (MFI) were exported as an Excel file.
2. Graphs from the MFI values were generated using GraphPad Prism. For MALII binding, MFI data from Jurkat cells treated with Ac_5_Neu5Ac3F were normalized to control, DMSO-treated cells (100% binding) (Table 1). Dose-response curves were generated by transforming the SAMs concentrations to log-scale. The non-linear regression fit tool was selected (Log(agonist) vs. response – variable slope (four parameters) to generate the dose-response curves and the EC50 values.

**Table 1:** Triplicate values of normalized MALII binding, with raw MFI values normalized to DMSO

Concentration (μM)	DMSO			Ac_5_Neu5Ac3F		
	1	2	3	1	2	3
0	100.00	100.00	100.00	100.84	94.35	102.09
2	99.26	110.39	100.20	89.87	93.09	76.97
4	94.35	101.90	96.61	78.14	64.65	71.10
8	111.33	90.01	105.11	55.41	59.81	60.84
16	110.01	95.29	104.54	63.92	38.12	44.13
32	95.67	110.39	95.29	46.18	42.51	49.40
56	97.94	102.65	94.35	34.30	32.84	36.50
128	94.92	99.26	105.29	32.55	33.86	26.39
256	109.45	93.78	108.31	29.32	30.49	27.56

These values were used to generate Figure 3.

## Limitations

In principle, all mammalian cells and tissues have sialoglycan biosynthesis capacity and should tolerate engineering with SAMs. This makes sialome engineering with SAMs broadly applicable to a large panel of mammalian cell lines and primary cells. The dependency on the sialoglycan biosynthesis pathway, requires that functional modifications of SAMs need to be tolerated by the involved enzymes and that they compete with endogenous pools of sialic acids. Design of novel SAMs can be limited by this, but several potent SAMs have already been generated with different functionalities to select from for sialome engineering.^2^^,^^6^ An advantage of SAMs is that they can be readily added and removed to mammalian cell culture medium and thus their activity can be temporally controlled. However, there is a delay effect that needs to be considered, because from the point of addition and removal the *de novo* sialoglycan biosynthesis and turnover rates determine the speed of incorporation or inhibition. Also, it takes a certain time (ca. 24 h) to build and deplete an intracellular pool of SAMs. Thus, effective concentrations and timing will depend on the cell-type specific sialoglycan biosynthesis and turnover rates. Additionally, SAM-based engineering utilizes the sialome generated by a cell and is therefore limited to the specific sialoglycans, glycoconjugates and glycoprotein/lipid of that cell. This could potentially influence the recognition by GBPs with specificity for linkage and glycoconjugate types. The nature of biosynthesized sialoglycans determines the products of the on-cell synthesis of high-affinity Siglec ligands that may thus produce different results in different cell types.

## Troubleshooting

### Problem 1

After treatment, there seems to be no effect of SAMs on the mammalian sialome.

### Potential solution 1

The cell type and concentration and incubation time can have large effects on the efficacy of SAMs. We advise to make a concentration range (e.g., 0–500 μM) and to incubate the cells for at least 48 h to give enough time for metabolic incorporation (step 1). For novel SAMs, it could be that they are not tolerized by the sialoglycan biosynthesis pathway. Known, validated inhibitory or incorporative SAMs should be included as positive controls.

### Problem 2

GBP (including Siglec Fc proteins) binding to cells is low/weak.

### Potential solution 2

This can have multiple reasons, for example that the used cell type does not produce the respective sialoglycan epitope needed for GBP binding or that the pre-complexing ratio of recombinant Siglec Fc protein to anti-human IgG detection antibody was not ideal. Alternatively, GBPs such as soluble recombinant Siglec Fc proteins can lose activity when stored at −20°C or −80°C for longer times and during repeated freeze-thaw cycles. GBP can differ in their binding potential between suppliers and batches. Concentrations should be evaluated prior to subsequent use. We recommend to aliquot such GBPs and to use them within 6 months after reconstitution and to avoid freeze-thaw cycles. Note that some GBPs require divalent ions (Ca^2+^ and Mg^2+^) for glycan binding.

### Problem 3

The click reaction is not efficient (steps 15, 23, 36).

### Potential solution 3

This protocol makes use of the CuAAC reaction. Excess protein in the reaction buffer, for example BSA from blocking steps, lowers the efficacy of the reaction. We recommend thoroughly washing samples with 1× PBS before the click reaction to remove all free protein from blocking or staining steps. Letting the reaction proceed at 37°C for an additional 15–30 min may also enhance efficacy. Alkyne- and azide-biotin conjugates can lose activity during freezing-thawing and should be aliquoted to avoid multiple freeze-thaw cycles.

### Problem 4

Cells shift in flow cytometry data analysis in FSC/SSC after click reaction (step 23, 41).

### Potential solution 4

The copper and ascorbate in the click buffer generate ROS that can damage living cells. The longer the reaction proceeds, the more severe the effects on cell viability. We recommend keeping the click reaction short, to fix the cells before clicking, or if needed to add ROS-chelating agents (e.g., L-Histidine) to the reaction to reduce the impact on viability. Alternative, copper-free click methods exist, e.g., based on dibenzocyclooctyne (DBCO), that could be applied if cell viability is of importance for the experiment.

## Resource availability

### Lead contact

Further information and requests for resources and reagents should be directed to and will be fulfilled by the lead contact, Thomas J. Boltje, thomas.boltje@ru.nl.

### Materials availability

This study did not generate new materials.

## Data Availability

The published article includes all datasets generated or analyzed during this study.
